# Supplementary material for: Structural basis of spike RBM-specific human antibodies counteracting broad SARS-CoV-2 variants
Source: Commun Biol. 2023 Apr 11;6:395. doi: 10.1038/s42003-023-04782-6 (PMC10088672; doi:10.1038/s42003-023-04782-6)
Supplement: Supplementary file 2 — Supplementary Information [file 42003_2023_4782_MOESM2_ESM.pdf]

Supplementary Information

Structural basis of spike RBM-specific human antibodies counteracting broad SARS-CoV-2 variants

Kiyomi Shitaoka[1,#], Akifumi Higashiura[2,#], Yohei Kawano[1,#], Akima Yamamoto[2], Yoko Mizoguchi[3], Takao Hashiguchi[4], Norihisa Nishimichi[5], Shiyu Huang[1], Ayano Ito[1], Shun Ohki[1], Miyuki Kanda[6], Tomohiro Taniguchi[7], Rin Yoshizato[1], Hitoshi Azuma[1], Yasuo Kitajima[1], Yasuyuki Yokosaki[5], Satoshi Okada[3], Takemasa Sakaguchi[2], Tomoharu Yasuda[1†]

[1] Department of Immunology, Graduate School of Biomedical and Health Sciences, Hiroshima University, Hiroshima, 734-8551, Japan

[2] Department of Virology, Graduate School of Biomedical and Health Sciences, Hiroshima University, Hiroshima, 734-8551, Japan

[3] Department of Pediatrics, Graduate School of Biomedical and Health Sciences, Hiroshima University, Hiroshima, 734-8551, Japan

[4] Laboratory of Medical Virology, Institute for Frontier Life and Medical Sciences, Kyoto University, Kyoto 606-8507, Japan.

[5] Integrin-Matrix Biomedical Science, Translational Research Center, Hiroshima University, Hiroshima, 734-8551, Japan.

[6] Collaborative laboratory of Liquid Biopsy, Graduate School of Biomedical and Health Sciences, Hiroshima University, Hiroshima, 734-8551, Japan

[7] Division of General Internal Medicine and Infectious Diseases, Hiroshima Prefectural Hospital, Hiroshima, 734-8530

[#] These authors contributed equally to this work.

[†] Corresponding author. e-mail: yasudat@hiroshima-u.ac.jp

Correspondence:
Tomoharu Yasuda, Ph.D.
Affiliations: Department of Immunology, Graduate School of Biomedical and Health Sciences, Hiroshima University.
Mailing address: 1-2-3 Kasumi, Minami-ku, Hiroshima, 734-8551, Japan.
Email: yasudat@hiroshima-u.ac.jp
ORCID: 0000-0001-9997-6852

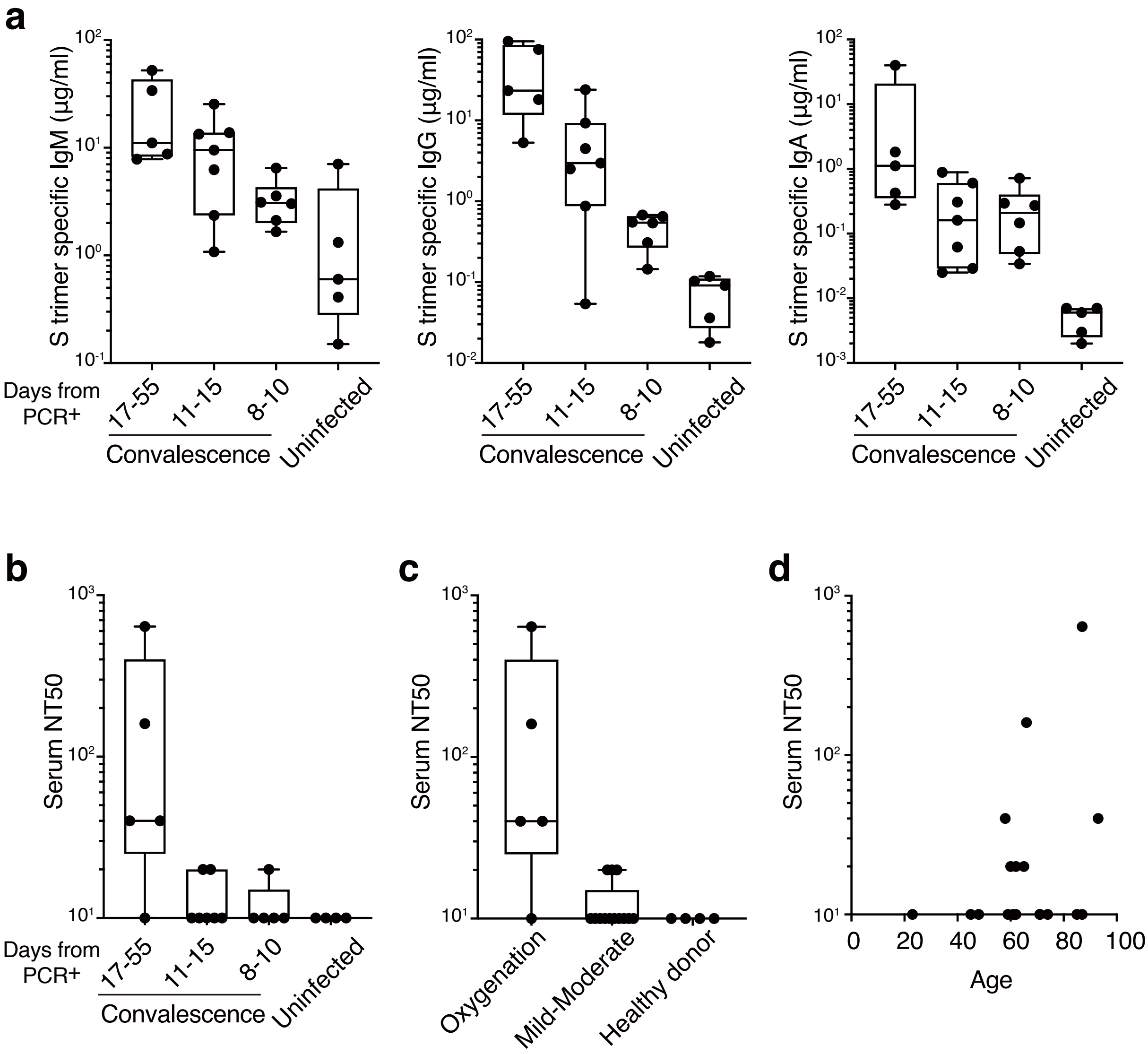

Supplementary Fig. 1 COVID-19 convalescent individuals after the long hospitalization show higher S trimer-specific antibodies and neutralization activity against SARS-CoV-2. **a**, The S trimer-specific serum IgM, IgG, and IgA levels were determined by ELISA. **b**, Serum neutralizing antibody titers of COVID-19 convalescent individuals subgrouped by hospitalization period from PCR result and uninfected healthy donors. The neutralizing activity of serum antibodies was evaluated by testing the blocking effect of authentic SARS-CoV-2 D614G virus infection to Vero cells. The 50% neutralization titer (NT_{50}) was determined using the half-maximal inhibitory concentration values. **c**, Serum neutralizing antibody titers of COVID-19 convalescent individuals subgrouped by the severity of the disease. **a-c**, Each box indicates the median and 25-75 percentile with min to max whiskers. **d**, Serum neutralizing antibody titer was plotted to the age of COVID-19 convalescent individuals.

a

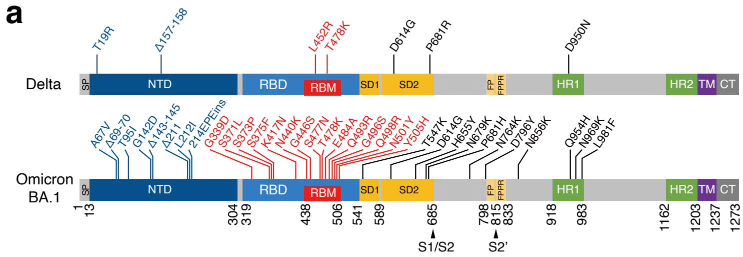

b

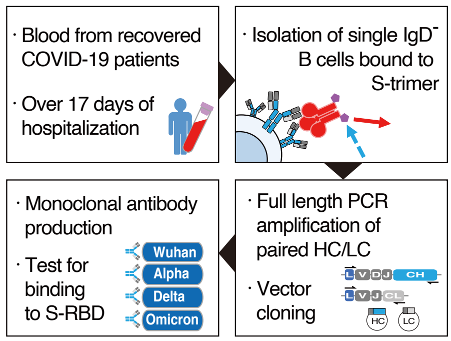

c

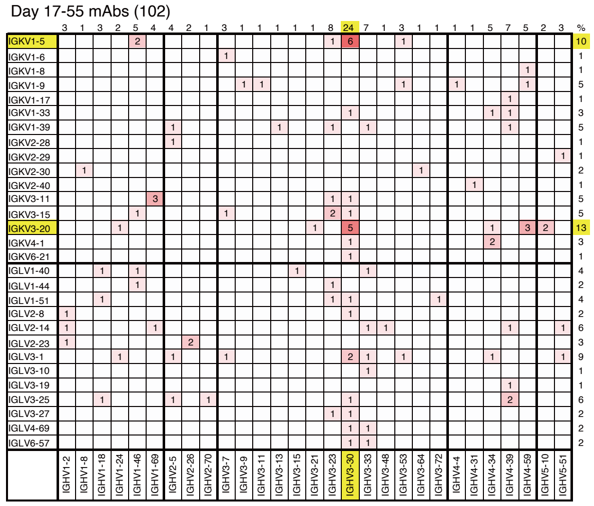

d

Authentic SARS-CoV-2 neutralization	IC_{50} (µg ml^{-1})		
	D614G (n = 3 or 4)	Delta (n = 2)	Omicron BA.1 (n = 3)
NCV1SG17	0.02 ± 0.01	1.09 ± 0.13	> 500
NCV1SG23	0.51 ± 0.22	5.61 ± 6.39	> 500
NCV2SG48	0.38 ± 0.24	0.20 ± 0.12	109 ± 55
NCV2SG53	0.06 ± 0.03	0.46 ± 0.38	> 500

e

Clone	IGH			
	Isotype	VH	CDR3	Mutation
NCV1SG17	IgG1	V4-39	CARLRYFDWFLDHW	4
NCV1SG23	IgG1	V1-46	CARDRRNETDCTGGVCRLNWFDPW	5
NCV2SG48	IgG1	V3-53	CARDLVVYGLDYW	6
NCV2SG53	IgG1	V3-11	CAREFDLTKIIMVPPYW	7

f

Clone	IGL			
	Isotype	VL	CDR3	Mutation
NCV1SG17	Igλ	V3-19	CNSRDSSGNHPF	1
NCV1SG23	Igλ	V1-40	CQSYDNILSCRVF	5
NCV2SG48	Igκ	V1-9	CQHLNSNPPITF	7
NCV2SG53	Igκ	V1-9	CQQLKSYPLTF	5

Supplementary Fig. 2 Production of S-trimer-specific mAbs and neutralization of authentic SARS-CoV-2. a, Mutational landscape of Delta (B.1.617.2) and Omicron (B.1.1.529, BA.1) are shown in the structure of SARS-CoV-2 spike protein with domains and cleavage sites. **b**, Strategy for isolation of S-trimer-specific mAbs from single-cell sorted Ig-switched B cells in the blood of COVID-19 convalescent individuals. **c**, The variable (V) gene frequencies for paired heavy (*x-axes*) and light (*y-axes*) chains of isolated S-trimer-specific mAbs. Numbers of identified V genes in 102 mAbs from 5 donors of day 17-55 hospitalization period are summarized with red color indication. The percent frequency of V genes is shown on the top and right of each panel for VH and VL, respectively. V genes account for over 10% and are highlighted in *yellow*. **d**, The IC_{50} neutralization values of each mAb to indicated authentic SARS-CoV-2 viruses. Mean ± SD from the indicated number of independent experiments is shown. **e** and **f**, Isotype, VH and VL genes, CDR3 amino acid sequence, and the number of amino acid mutations of indicated neutralizing mAbs are shown.

a

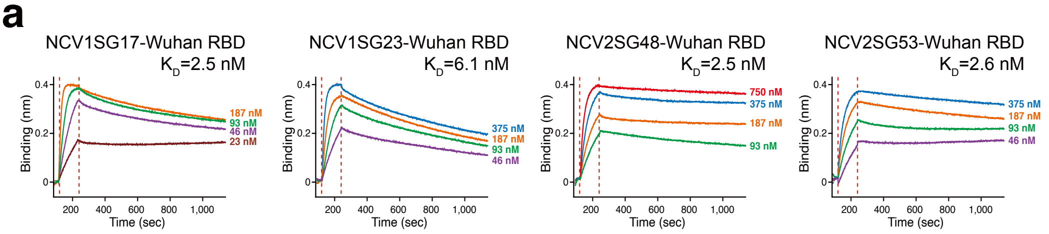

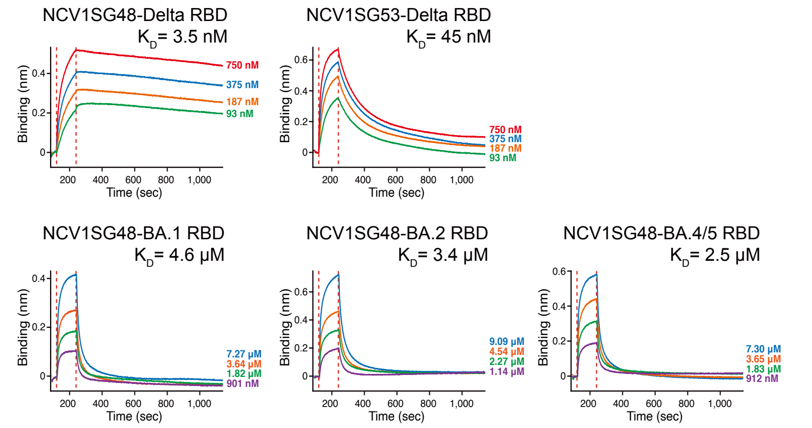

mAb	S RBD	KD (nM)	ka (1/Ms) $x10^5$	kd (1/s) $x10^{-4}$
NCV1SG17	Wuhan	2.5	3.3	8.0
NCV1SG23	Wuhan	6.1	1.6	9.9
NCV2SG48	Wuhan	2.5	0.60	1.5
	Delta	3.5	0.50	1.7
	Omicron BA.1	4,641	0.10	457
	Omicron BA.2	3,384	0.09	290
	Omicron BA.4/5	2,506	0.15	364
NCV2SG53	Wuhan	2.6	0.85	2.2
	Delta	45	1.2	52

b

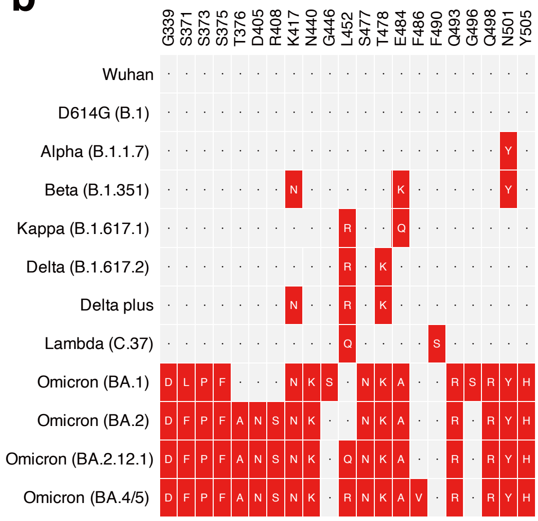

c

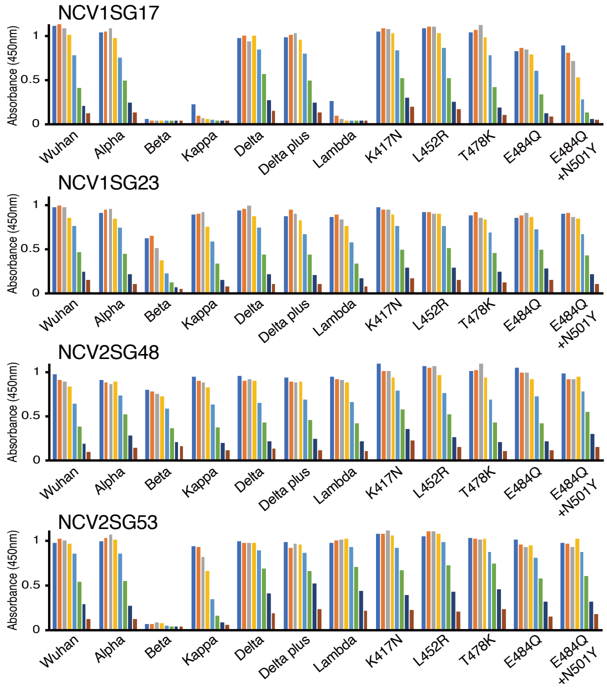

Supplementary Fig. 3 Binding of neutralizing Abs to various SARS-CoV-2 RBD mutants and affinity to RBD. a, Biolayer interferometry results. The binding affinity of NCV1SG17, NCV1SG23, NCV2SG48, and NCV2SG53 against RBD proteins of Wuhan, Delta, and Omicron variants. Binding kinetics were measured for four different concentrations of the antigen and evaluated using a 1:1 binding model. **b**, The matrix represents amino acid substitutions present in RBD of indicated SARS-CoV-2 variants. The name of SARS-CoV-2 variant is given on the *y-axis* and the position and amino acid replacement (single letter code) in each strain is given on the *x-axis*. **c**, The binding of neutralizing mAbs with S RBD protein of indicated variants or point mutants is determined by ELISA by three-fold serial dilutions.

a

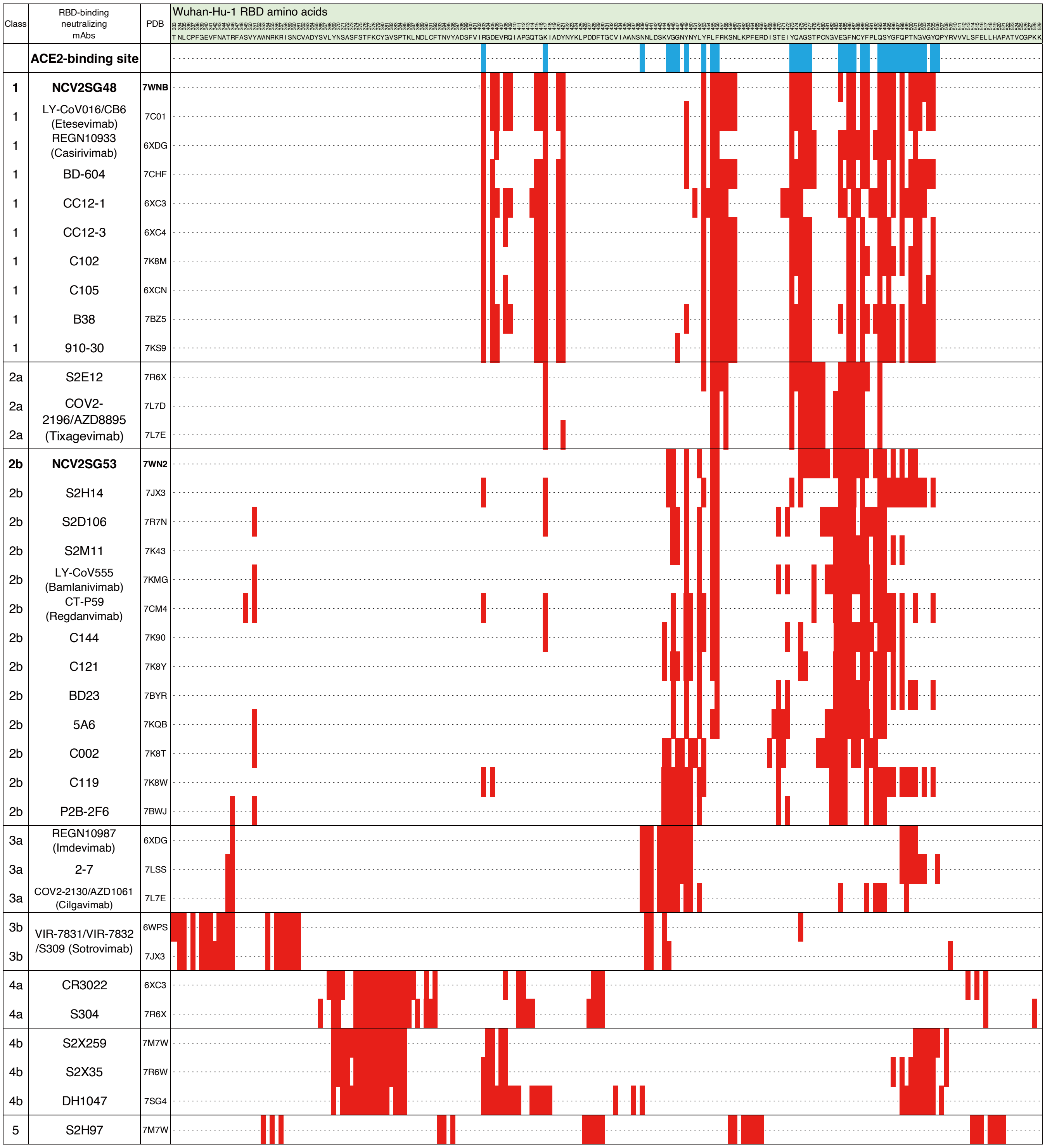

b

Class	RBD-binding neutralizing mAbs	Alpha B.1.1.7	Beta B.1.351	Delta B.1.617.2	Omicron BA.1	Omicron BA.2	Omicron BA.2.12.1	Omicron BA.4/5
**1**	**NCV2SG48**	+++	+++	+++	+++	++	+++	+++
1	REGN10933 (Casirivimab)	+++	-	+++	-	-	-	-
1	LY-CoV016 (Etesevimab)	+++	-	+++	-	-	-	-
2a	AZD8895 (Tixagevimab)	+++	++	+++	-	-	-	-
**2b**	**NCV2SG53**	+++	-	+++	-	-	-	-
2b	LY-CoV555 (Bamlanivimab)	+++	-	-	-	-	-	-
2b	CT-P59 (Regdanvimab)	na	na	++	-	-	na	-
3a	REGN10987 (Imdevimab)	+++	+++	+++	-	-	-	-
3a	AZD1061 (Cilgavimab)	+++	+++	+++	++	+++	+++	++
3b	Vir-7831 (Sotrovimab)	+++	+++	+++	+++	++	++	++

Supplementary Fig. 4 Epitope map and relative neutralization activity of monoclonal antibodies. a, Amino acid residues 333-529 of SARS-CoV-2 Wuhan-Hu-1 RBD are shown. ACE2 binding sites on RBD are shown in blue. The epitope footprints of NCV2SG48, NCV2SG53, and EUA mAbs on RBD determined by X-ray crystallography are shown in red. The S2H97 is categorized as class 5 because of a different binding pattern from other neutralizing mAb classes. **b**, The retained neutralization levels of mAbs against indicated SARS-CoV-2 variants compared to Wuhan-Hu-1 or D614G parental strain are summarized from this study and previous reports of pseudovirus assay [1-7]. +++, less than 15-fold reduction; ++, 15-100-fold reduction; -, more than 100-fold reduction or no activity; na, not applicable.

a

Neutralizing Antibody	HC CDR 1	HC CDR 2	HC DE-loop	HC CDR 3	HC Total	LC CDR 1	LC CDR 2	LC DE-loop	LC CDR 3	LC Total	HC+LC Total
	Number of hydrogen bonds with RBD (including water-mediated bonds)										
NCV2SG48	7	15(7)	0	4	26(7)	8(4)	0	0	6(2)	14(6)	40(13)
NCV2SG53	2	6(1)	3(1)	1	12(2)	2(1)	0	0	4(4)	6(5)	18(7)

b

Class	RBD mAb	PDB ID	Resolution (A)	Number of hydrogen bonds HC	LC	HC+LC
1	**NCV2SG48**	**7WNB**	**2.18**	**19**	**8**	**27**
	CB6	7C01	2.88	20	5	25
	BD-604	7CHF	2.67	16	9	25
	CC12.1	6XC3	2.70	16	13	29
	CC12.3	6XC4	2.34	17	4	21
	B38	7BZ5	1.84	17	13	30
2a	S2E12	7R6X	2.95	7	1	8
	AZD8895	7L7D	2.5	8	3	11
2b	**NCV2SG53**	**7WN2**	**2.53**	**8**	**5**	**13**
	S2H14	7JX3	2.65	13	5	18
	S2M11	7K43	2.60	8	0	8
	LY-COV555	7KMG	2.16	12	4	16
	CT-P59	7CM4	2.71	13	2	15
	5A6	7KQB	2.42	5	4	9
	P2B-2F6	7BWJ	2.85	7	2	9
3b	S309	7JX3	2.65	9	4	13
4a	S304	7JX3	2.65	6	10	16
4b	S2X259	7M7W	2.65	9	7	16
	S2X35	7R6W	1.83	13	5	18

c

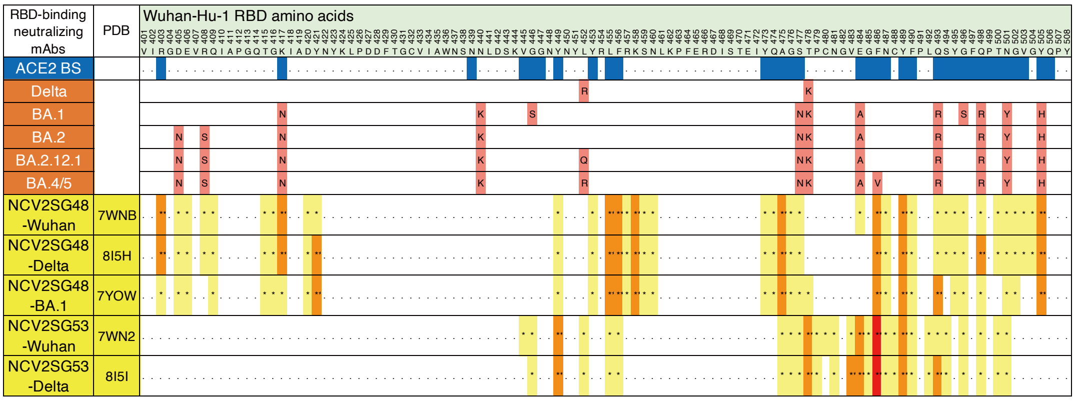

Supplementary Fig. 5 Hydrogen bonds of NCV2SG48 and NCV2SG53 with SARS-CoV-2 RBD. a, Summary of the hydrogen bond number formed at the interface of NCV2SG48 or NCV2SG53 with Wuhan-Hu-1 RBD. The number of water-mediated hydrogen bonds is indicated by parentheses. **b**, Summary of the hydrogen bond number formed at the interface of reported neutralizing mAb with Wuhan-Hu-1 RBD. PDB, protein data bank; HC, heavy chain; LC, light chain. **c**, Epitope map of neutralizing antibodies on SARS-CoV-2 RBD variants. Residues 401-508 of SARS-CoV-2 Wuhan-Hu-1 RBD are shown. ACE2 binding sites are shown in blue. The epitope of NCV2SG48 and NCV2SG53 determined by X-ray crystallography are shown in highlighted asterisks. Residues with buried surface area (BSA) values greater than 0 as calculated by PISA are identified as binding interfaces. 0<BSA<50 is indicated in yellow, 50≦BSA<100 in orange, and BSA≧100 in red.

a

Antibody	SARS-CoV-2 virus (pfu)	Number of escape clone
NCV1SG17	2.2 x 10^4	0
	1.2 x 10^5	1 (EM-17-1)
	5.6 x 10^4	4 (EM-17-2)
	1.4 x 10^5	1 (EM-17-3)
Total	3.4 x 10^5	6
NCV1SG23	2.2 x 10^2	0
	1.2 x 10^3	0
	5.6 x 10^2	0
	3.7 x 10^3	0
	3.1 x 10^4	0
	1.3 x 10^4	0
	1.5 x 10^4	0
Total	6.4 x 10^4	0
NCV2SG48	2.2 x 10^2	0
	1.2 x 10^3	0
	5.6 x 10^2	0
	7.9 x 10^2	0
	2.6 x 10^4	0
	4.3 x 10^4	0
	5.1 x 10^4	0
Total	1.2 x 10^5	0
NCV2SG53	2.2 x 10^3	0
	1.2 x 10^4	0
	5.6 x 10^3	0
	2.8 x 10^4	0
	9.8 x 10^4	1 (EM53-1)
	1.1 x 10^5	1 (EM53-2)
	1.4 x 10^5	3 (EM53-4)
Total	3.6 x 10^5	5

b

Escape clone	Identified mutation	Neutralization, MIC (µg/ml)			
		NCV1 SG17	NCV1 SG23	NCV2 SG48	NCV2 SG53
EM-17-1	S494P	> 100	< 2.5	< 2.5	50-100
EM-17-2	S494P	> 100	< 2.5	< 2.5	50-100
EM-17-3	S494P	> 100	< 2.5	< 2.5	50-100
EM-53-1	E484D	> 100	< 2.5	< 2.5	> 100
EM-53-2	G485D	> 100	< 2.5	< 2.5	> 100
EM-53-4	G485R	> 100	< 2.5	< 2.5	> 100
Parental SARS-CoV-2		< 2.5	< 2.5	< 2.5	< 2.5

Supplementary Fig. 6 Screening of escape mutants and neutralization results. a, Escape mutant screening was performed under the presence of NCV1SG17, NCV1SG23, NCV2SG48, or NCV2SG53 neutralization mAbs. Independent screening was repeated four to seven times for each mAb. **b**, Isolated escape mutants were sequenced to identify specific mutations and used for neutralization assay with a panel of neutralizing antibodies. MIC: minimum inhibitory concentration.

Supplementary Table 1. COVID-19 convalescent blood donor information.

Donor ID	Sex	Age	Date PCR positive	Severity	Date blood collection	Days from PCR+	SARS-COV2 Type
HD01	M	46	NA	Uninfected	2020/10/22	NA	NA
HD02	M	44	NA	Uninfected	2020/12/8	NA	NA
HD03	M	37	NA	Uninfected	2020/10/22	NA	NA
HD04	M	37	NA	Uninfected	2020/12/8	NA	NA
HD05	F	36	NA	Uninfected	2020/5/12	NA	NA
HD06	F	62	NA	Uninfected	2021/4/15	NA	NA
HD07	F	54	NA	Uninfected	2021/4/15	NA	NA
HD08	F	49	NA	Uninfected	2021/4/15	NA	NA
HD09	F	48	NA	Uninfected	2021/4/15	NA	NA
NCV01	F	93	2020/4/14	Oxygenation	2020/6/8	55	D614G
NCV02	F	87	2020/4/14	Oxygenation	2020/6/8	55	D614G
NCV03	M	45	2020/8/11	Mild/Moderate	2020/8/20	9	D614G
NCV04	M	58	2020/8/5	Oxygenation	2020/8/28	23	D614G
NCV05	F	65	2020/12/3	Mild/Moderate	2020/12/13	10	D614G
NCV06	M	61	2020/12/7	Mild/Moderate	2020/12/16	9	D614G
NCV07	F	66	2020/12/3	Oxygenation	2020/12/20	17	D614G
NCV08	M	71	2020/12/3	Oxygenation	2020/12/20	17	D614G
NCV09	M	71	2020/12/8	Mild/Moderate	2020/12/21	13	D614G
NCV10	F	74	2020/12/9	Mild/Moderate	2020/12/21	12	D614G
NCV11	F	48	2020/12/18	Mild/Moderate	2020/12/28	10	D614G
NCV12	F	23	2020/12/20	Mild/Moderate	2020/12/28	8	D614G
NCV13	F	87	2020/12/29	Mild/Moderate	2021/1/11	13	D614G
NCV14	F	59	2020/12/31	Mild/Moderate	2021/1/11	11	D614G
NCV15	F	62	2021/1/7	Mild/Moderate	2021/1/17	10	D614G
NCV16	M	62	2021/1/6	Mild/Moderate	2021/1/17	11	D614G
NCV17	F	60	2021/1/8	Mild/Moderate	2021/1/23	15	D614G
NCV18	F	85	2021/1/12	Mild/Moderate	2021/1/23	11	D614G

Supplementary Table 2. RBD binding interface area of neutralizing antibodies.

The interface area of Fab and the HC/LC occupancy of indicated mAbs with Wuhan-Hu-1 RBD was calculated.

RBD mAb	Class	Interface area (Å)	% of interface area		PDB	Resolution (A)
		Fab	HC	LC		
**NCV2SG48**	**1**	**1200.4**	**66.5**	**35.6**	**7WNB**	**2.18**
LY-CoV016/CB6 (Etesevimab)	1	1074.4	68.7	31.9	7C01	2.88
REGN10933 (Casirivimab)	1	894.2	83.6	20.1	6XDG	3.90
BD-604	1	1112.6	66.6	35.5	7CHF	2.67
CC12.1	1	1003.5	66.8	33.6	6XC3	2.70
CC12.3	1	877	80	20	6XC4	2.34
C102	1	944.5	75.2	25.9	7K8M	3.20
C105	1	852.6	73.9	27.1	6XCN	3.66
B38	1	1203.1	59.4	40.8	7BZ5	1.84
910-30	1	1121.2	60.0	41.9	7KS9	4.75
S2E12	2a	721.6	73.2	30.9	7R6X	2.95
COV2-2196/AZD8895 (Tixagevimab)	2a	406.2	72.8	30.0	7L7D	2.50
		636.6	72.3	32.5	7L7E	3.00
**NCV2SG53**	**2b**	**858.7**	**73.2**	**32.8**	**7WN2**	**2.53**
S2H14	2b	867.3	58.9	46.8	7JX3	2.65
S2D106	2b	758.8	76.2	28.5	7R7N	3.95
S2M11	2b	640.4	95.5	8.7	7K43	2.60
LY-CoV555 (Bamlanivimab)	2b	789.8	74.7	30.5	7KMG	2.16
CT-P59 (Regdanvimab)	2b	737.3	90.8	9.9	7CM4	2.71
C144	2b	761.7	91.6	12.4	7K90	3.24
C121	2b	760.6	96.0	7.0	7K8Y	4.40
BD23	2b	760.4	96.3	4.1	7BYR	3.84
5A6	2b	884.5	79.7	31.6	7KQB	2.42
C002	2b	846.5	84.6	19.6	7K8T	3.40
C119	2b	868.9	64.5	37.1	7K8W	3.60
P2B-2F6	2b	547.3	81.3	23.0	7BWJ	2.85
REGN10987 (Imdevimab)	3a	584	84.8	18.7	6XDG	3.90
2-7	3a	746.4	59.7	45.6	7LSS	3.72
COV2-2130/AZD1061 (Cilgavimab)	3a	736.1	58.5	43.5	7L7E	3.00
VIR-7831/VIR-7832 /S309 (Sotrovimab)	3b	763.0	83.0	21.8	6WPS	3.10
		747.5	85.0	20.6	7JX3	2.65
CR3022	4a	906.6	64.5	40.1	6XC3	2.70
S304	4a	837.2	59.7	44.7	7R6X	2.95
S2X259	4b	954.2	70.0	33.0	7M7W	2.65
S2X35	4b	916.2	73.9	30.5	7R6W	1.83
DH1047	4b	1280.0	59.9	44.3	7SG4	3.43
S2H97	5	795.4	71.5	34.2	7M7W	2.65

Supplementary References

1 Planas, D. *et al.* Reduced sensitivity of SARS-CoV-2 variant Delta to antibody neutralization. *Nature* **596**, 276-280, doi:10.1038/s41586-021-03777-9 (2021).

2 Wang, P. *et al.* Antibody resistance of SARS-CoV-2 variants B.1.351 and B.1.1.7. *Nature* **593**, 130-135, doi:10.1038/s41586-021-03398-2 (2021).

3 Cameroni, E. *et al.* Broadly neutralizing antibodies overcome SARS-CoV-2 Omicron antigenic shift. *Nature*, doi:10.1038/s41586-021-04386-2 (2021).

4 Planas, D. *et al.* Considerable escape of SARS-CoV-2 Omicron to antibody neutralization. *Nature*, doi:10.1038/s41586-021-04389-z (2021).

5 VanBlargan, L. A. *et al.* An infectious SARS-CoV-2 B.1.1.529 Omicron virus escapes neutralization by therapeutic monoclonal antibodies. *Nat Med* **28**, 490-495, doi:10.1038/s41591-021-01678-y (2022).

6 Cox, M. *et al.* SARS-CoV-2 variant evasion of monoclonal antibodies based on in vitro studies. *Nat Rev Microbiol* **21**, 112-124, doi:10.1038/s41579-022-00809-7 (2023).

7 Yamasoba, D. *et al.* Neutralisation sensitivity of SARS-CoV-2 omicron subvariants to therapeutic monoclonal antibodies. *Lancet Infect Dis* **22**, 942-943, doi:10.1016/S1473-3099(22)00365-6 (2022).
