# Supplementary material for: Structural basis of spike RBM-specific human antibodies counteracting broad SARS-CoV-2 variants
Source: Commun Biol. 2023 Apr 11;6:395. doi: 10.1038/s42003-023-04782-6 (PMC10088672; doi:10.1038/s42003-023-04782-6)
Supplement: Supplementary file 3 — Description of Additional Supplementary Files [file 42003_2023_4782_MOESM3_ESM.pdf]

Description of Additional Supplementary Files

File name: Supplementary Data 1

Description: The numerical source data and replicate data for Figure 1, a, d, e, and f.

File name: Supplementary Data 2

Description: The numerical source data and replicate data for Figure 2a.

File name: Supplementary Data 3

Description: The numerical source data and replicate data for Figure 4e.

File name: Supplementary Data 4

Description: The summary of VH and VL usage of isolated mAbs from donor NCV1, 2, 4, 7, and 8.
