# Supplementary material for: Structural basis of spike RBM-specific human antibodies counteracting broad SARS-CoV-2 variants
Source: Commun Biol. 2023 Apr 11;6:395. doi: 10.1038/s42003-023-04782-6 (PMC10088672; doi:10.1038/s42003-023-04782-6)
Supplement: Supplementary file 8 — Reporting Summary [file 42003_2023_4782_MOESM8_ESM.pdf]

nature portfolio

Corresponding author(s): Tomoharu Yasuda

Last updated by author(s): Feb 23, 2023

Reporting Summary

Nature Portfolio wishes to improve the reproducibility of the work that we publish. This form provides structure for consistency and transparency in reporting. For further information on Nature Portfolio policies, see our Editorial Policies and the Editorial Policy Checklist.

Statistics

For all statistical analyses, confirm that the following items are present in the figure legend, table legend, main text, or Methods section.

n/a	Confirmed	
☐	☒	The exact sample size (n) for each experimental group/condition, given as a discrete number and unit of measurement
☐	☒	A statement on whether measurements were taken from distinct samples or whether the same sample was measured repeatedly
☐	☒	The statistical test(s) used AND whether they are one- or two-sided<br>*Only common tests should be described solely by name; describe more complex techniques in the Methods section.*
☒	☐	A description of all covariates tested
☒	☐	A description of any assumptions or corrections, such as tests of normality and adjustment for multiple comparisons
☒	☐	A full description of the statistical parameters including central tendency (e.g. means) or other basic estimates (e.g. regression coefficient) AND variation (e.g. standard deviation) or associated estimates of uncertainty (e.g. confidence intervals)
☒	☐	For null hypothesis testing, the test statistic (e.g. F, t, r) with confidence intervals, effect sizes, degrees of freedom and P value noted<br>*Give P values as exact values whenever suitable.*
☒	☐	For Bayesian analysis, information on the choice of priors and Markov chain Monte Carlo settings
☒	☐	For hierarchical and complex designs, identification of the appropriate level for tests and full reporting of outcomes
☒	☐	Estimates of effect sizes (e.g. Cohen's d, Pearson's r), indicating how they were calculated

Our web collection on statistics for biologists contains articles on many of the points above.

Software and code

Policy information about availability of computer code

Data collection	ELISA analysis, iMark Microplate Reader (BIO-RAD); FACS analysis, BD LSRFortessa (BD Biosciences), CytoFLEX S (Beckman Coulter); Single cell sorting, Special Order System BD FACSAria II (BD Biosciences); Luciferase assay, ARVO X13 (Perkin Elmer); Chromatography, NGC Chromatography Systems (BIO-RAD)
Data analysis	ELISA analysis, MPM 6 (BIO-RAD); FACS analysis, BD FACSDiva (v8.0.2, BD Biosciences), CytExpert (v2.4, BECKMAN-COULTER), or FlowJo (v10.8.1, BD Biosciences); Luciferase assay, Perkin Elmer 2030 Workstation (Perkin Elmer), Statistical analysis, Prism 9.0 (GraphPad); FACS analysis, FlowJo v10.8.1 (BD Biosciences); Chromatography, ChromLab v6 (BIO-RAD); X-ray data analysis, XDS (Kabsch et al., 2010); Structure analysis, CCP4 7.1 (Winn et al., 2011); Structure analysis, PHENIX version 1.19 (Liebschner et al., 2019); Molecular visualization, The PyMOL Molecular Graphics System Version 2.4.0. (Schrödinger, LLC)

For manuscripts utilizing custom algorithms or software that are central to the research but not yet described in published literature, software must be made available to editors and reviewers. We strongly encourage code deposition in a community repository (e.g. GitHub). See the Nature Portfolio guidelines for submitting code & software for further information.

Data

Policy information about availability of data

All manuscripts must include a data availability statement. This statement should provide the following information, where applicable:

- Accession codes, unique identifiers, or web links for publicly available datasets
- A description of any restrictions on data availability
- For clinical datasets or third party data, please ensure that the statement adheres to our policy

The structure of SARS-CoV-2 RBD in complex with NCV2SG48 and NCV2SG53 Fabs has been deposited in the Protein Data Bank as the PDB ID:7WNB and 7WN2, respectively. Source data for figures are provided in the paper or available from the corresponding author upon request. Monoclonal antibodies will be available upon request for research use. Human subjects will not be available for researchers who are not listed in the human study protocol.

Human research participants

Policy information about studies involving human research participants and Sex and Gender in Research.

Reporting on sex and gender	We properly used terms of sex/gender. Finding does not distinguish sex difference. Sex was based on self-reporting or clinical record.
Population characteristics	The covariate-relevant population characteristics of human research participants are summarized in Supplementary Table 1.
Recruitment	Participants were recruited based on patients at collaborating hospitals, announcement, laboratory members, ore related persons.
Ethics oversight	The Ethical Committee for Epidemiology of Hiroshima University.

Note that full information on the approval of the study protocol must also be provided in the manuscript.

Field-specific reporting

Please select the one below that is the best fit for your research. If you are not sure, read the appropriate sections before making your selection.

☒ Life sciences ☐ Behavioural & social sciences ☐ Ecological, evolutionary & environmental sciences

For a reference copy of the document with all sections, see nature.com/documents/nr-reporting-summary-flat.pdf

Life sciences study design

All studies must disclose on these points even when the disclosure is negative.

Sample size	Sample size was determined to include more than two blood donors who show high level of serum antibody neutralizing Omicron variant.
Data exclusions	No data were excluded from the analysis.
Replication	We confirmed reproducibility of all presented data more than two times.
Randomization	We used all human subjects which we could obtain. Therefore, arbitrary allocation of human subjects was impossible.
Blinding	Sample preparation of human subjects, FACS sorting, monoclonal antibody generation, and characterization of monoclonal antibodies were all performed after the allocation of random IDs.

Reporting for specific materials, systems and methods

We require information from authors about some types of materials, experimental systems and methods used in many studies. Here, indicate whether each material, system or method listed is relevant to your study. If you are not sure if a list item applies to your research, read the appropriate section before selecting a response.

Materials & experimental systems

n/a	Involved in the study
☐	☒ Antibodies
☐	☒ Eukaryotic cell lines
☒	☐ Palaeontology and archaeology
☒	☐ Animals and other organisms
☒	☐ Clinical data
☒	☐ Dual use research of concern

Methods

n/a	Involved in the study
☒	☐ ChIP-seq
☒	☐ Flow cytometry
☒	☐ MRI-based neuroimaging

Antibodies

Antibodies used	FcX blocking antibodies (BioLegend, #4422302); CD19-APC-Cy7 (BioLegend, #302217); IgD-FITC (BioLegend, #348206); Monoclonal antibodies generated from human subjects.
Validation	Validation of commercial antibodies are all available from company website.

Eukaryotic cell lines

Policy information about cell lines and Sex and Gender in Research

Cell line source(s)	Expi293 cells are provided from Thermo Fisher Scientific. 293T cells are from human embryonic kidney.
Authentication	We purchased cell lines from company.
Mycoplasma contamination	Cell lines were tested negative for mycoplasma contamination.
Commonly misidentified lines (See ICLAC register)	None.
